# “What Is Right for Me, Is Not Necessarily Right for You”: The Endogenous Factors Influencing Nonparticipation in Medical Assistance in Dying

**DOI:** 10.1177/10497323211008843

**Published:** 2021-05-03

**Authors:** Janine Brown, Donna Goodridge, Lilian Thorpe, Alexander Crizzle

**Affiliations:** 1 University of Saskatchewan, Saskatoon, Saskatchewan, Canada; 2 University of Regina, Saskatoon, Saskatchewan, Canada

**Keywords:** medical assistance in dying, conscience objection, nonparticipation, physicians, nurse practitioners, decision-making, qualitative, interpretive description, Canada

## Abstract

Access to medical assistance in dying (MAID) is influenced by legislation, health care providers (HCPs), the number of patient requests, and the patients’ locations. This research explored the factors that influenced HCPs’ nonparticipation in formal MAID processes and their needs to support this emerging practice area. Using an interpretive description methodology, we interviewed 17 physicians and 18 nurse practitioners who identified as non-participators in formal MAID processes. Nonparticipation was influenced by their (a) previous personal and professional experiences, (b) comfort with death, (c) conceptualization of duty, (d) preferred end-of-life care approaches, (e) faith or spirituality beliefs, (f) self-accountability, (g) consideration of emotional labor, and (h) future emotional impact. They identified a need for clear care pathways and safe passage. Two separate yet overlapping concepts were identified, conscientious objection *to* and nonparticipation *in* MAID, and we discussed options to support the social contract of care between HCPs and patients.

Medical assistance in dying (MAID) is a legally available end-of-life (EOL) care option in Canada. Bill C-16, passed June 17, 2016, afforded an exemption from culpable homicide for physicians and nurse practitioners (NPs) who provide MAID to eligible Canadians (Government of Canada, 2016). This legislation stated eligible patients must (a) qualify for Canadian health services, (b) be mentally competent and at least 18 years and older, (c) have an irremediable and grievous medical condition, (d) request MAID voluntarily and without outside influence, and (e) provide informed consent. The Bill further outlined that an irremediable and grievous medical condition requires that (a) the illness, disease, or disability is serious and incurable; (b) the individual is in an advanced state of irreversible decline in capability; (c) that the illness, disease, or disability causes the individual enduring psychological or physical suffering that is intolerable to them and cannot be relieved through means they find acceptable; and (d) that, taken into all the medical circumstances, that the individual’s natural death has become reasonably foreseeable.

There are provincial, territorial, and regional variations to MAID programming (Pesut et al., 2020; Wiebe et al., 2020) attributed to differing health care delivery structures, geographical contexts, interests, resources, and performance indicators (Silvius et al., 2019). However, all provinces and territories must abide by the Canada Health Act, which requires patient care accessibility in health services delivery (Government of Canada, 1984). As such, patients must have “uniform,” “unprecluded,” and “unimpeded” access to legally available insured health care services (Health Canada, 2019, p. 9).

Since MAID was legalized, 13,946 Canadians have had a medically assisted death, and 80.6% of these deaths occurred in those aged 65 years and older (Health Canada, 2020). The number of MAID requests will likely increase as the Canadian population grows and the proportion of persons aged 65 years and older rises (Statistics Canada, 2019). Furthermore, increases in MAID requests at all ages is plausible as MAID becomes more widely accepted in Canada. These projected increases are substantiated as research from international regions with assisted dying reported an increase in the number of patients accessing care over time (Steck et al., 2013). Access to MAID varies throughout Canada (Brown et al., 2020; Oczkowski et al., 2020; Schiller, 2017). Individuals in rural and remote areas experience health care access challenges and poorer health outcomes (Greenburg et al., 2019), and so it is reasonable they will experience MAID access challenges as well. Thus, access to MAID is influenced not only by the availability of health care providers (HCPs) but also by the number of patients requesting MAID and the location of their residence.

This research was conducted between May and September 2019 in the Canadian province of Saskatchewan, with an approximate population of 1,170,000, and 38% of the population living in rural or remote areas (Statistics Canada, 2016). In December 2017, individual health regions merged into a single publicly funded provincial health authority and, in November 2018, formal MAID processes became the responsibility of a provincial MAID program (Bridges, 2019). The MAID program has a number of salaried employees, including one NP based in each of the two largest cities. Although program NPs perform MAID assessments and provisions, much of the clinical MAID work is provided on a case-by-case basis by NPs and physicians from diverse practice areas (e.g., family medicine, obstetrics, psychiatry, and anesthesia). Access to the MAID program was initially through a HCP-initiated referral, but since the provincial program’s development, patients, family members, or others may initiate referrals through the provincial Healthline.

There are approximately 2,600 licensed physicians (Saskatchewan Ministry of Health, 2019) and 267 registered NPs (Saskatchewan Registered Nurses Association, 2018) in Saskatchewan. These HCPs are essential to MAID access as they are the only two professional groups that can determine a patient’s eligibility for MAID and provide MAID. Since MAID legalization, Saskatchewan has reported 250 MAID deaths (Health Canada, 2020). According to the provincial MAID program, between November 2018 and February 2020, 35 (or 0.012%) physicians and NPs participated in either MAID assessments or MAID provisions, or both, with 17 of them participating in fewer than five instances (M. Fisher, personal communication, February 27, 2020). Canadian MAID assessors and providers characterized their participation as rewarding (Shaw et al., 2018) and as an honor, a privilege, and as a “life-transforming gift” (McKee & Sellick, 2018, p. e89). It allowed practicing whole-person care, supported emotional engagement with patients, and resulted in “personal and professional well-being [that] is gained from satisfaction and appreciation of living core values” (Beuthin et al., 2020, p. 3). However, participating HCPs also noted that the administrative demands of time, workload, “the learning curve,” geographical isolation, and lack of team support were sources of stress (McKee & Sellick, 2018, p. e89). Additional challenges for HCPs in participating in formal MAID processes included inadequate compensation, strained relationships with objecting colleagues, and sacrifices to personal time (Khoshnood et al., 2018). They also cited that working with institutions with a conscientious objection (CO), denying patients who did not qualify for MAID, working with family and friends through grief, and “feeling like they were on call all the time” complicated MAID provision (Shaw et al., 2018, p. e398).

There is emerging yet limited research exploring the motivations of those who do and do not participate in MAID. Oliphant and Frolic (2020) explored the factors that precipitated conscientious participation in MAID and highlighted that participants were motivated by their personal and professional values and identity, and influenced by their experience with death and dying and the organizational context where MAID occurs. Conversely, Bouthillier and Opatrny (2019) explored CO to MAID and determined that the majority of physicians used CO “as a mechanism to opt-out of medical aid in dying for a multitude of reasons other than religious or moral objections” (p. 1212).

Patient requests for MAID are subject to increase over time (Statistics Canada, 2019; Steck et al., 2013), and participating HCPs, who are essential to formal MAID processes, reported numerous practice rewards, challenges, and stressors (Beuthin et al., 2020; Khoshnood et al., 2018; McKee & Sellick, 2018; Shaw et al., 2018). Few HCPs in Saskatchewan participate in the formal MAID process and there is limited evidence on the participation of HCPs in this practice context. Thus, understanding the factors that influence HCPs’ nonparticipation in formal MAID processes is a high priority for research to support HCPs in this emerging practice area and patients’ care access. This research aims to identify the factors that influence physicians and NPs when deciding not to participate in the formal MAID processes of determining a patient’s eligibility for MAID and providing MAID and HCPs’ needs in this emerging practice area.

## Background

HCPs balance multiple considerations in their professional practices. HCPs work within what Kinnier et al. (2000) proposed are the moral values common within diverse societies. These common moral values include (a) a commitment to something greater than oneself; (b) self-respect with humility, self-discipline, and acceptance of responsibility; (c) respect and caring for others; and (d) care for other living things and the environment. Professional codes of ethics also guide HCPs’ practices. These codes include the virtues of compassion, honesty, humility, integrity, and prudence (Canadian Medical Association, 2018), as well as the values of safe and compassionate ethical care, the promotion of health and well-being, informed decision-making, and dignity, privacy, confidentiality, justice, and accountability (Canadian Nurses Association, 2017a). In addition, society expects that individuals, including HCPs, abide by federal, provincial, and municipal laws in their interactions with others. As HCPs contemplate their care provision within these professional and ethical constructs, and societal moral values and laws, there may be tension. Consequently, HCPs may not participate in the full range of legally available care or the care requested by a patient, resulting in uncertainty regarding HCPs’ obligations when responding to these requests (McLeod & Downie, 2014).

Bill C-14 expressly guaranteed HCPs’ freedom of conscience and religion (Government of Canada, 2016). Some HCPs may have a CO to MAID, which is nonparticipation based on “a particularly important subset of an agent’s ethical or religious beliefs—[or] *core* moral beliefs” (Wicclair, 2011, p. 4). Conscience is an essential component of ethical care (Lamb et al., 2017), and Askin and Bouchard (2018) articulated that freedom of conscience is doing what one feels *must* be done. Wicclair (2011) noted that CO views are placed along a continuum, ranging from where one’s conscience is morally binding to where one’s moral and ethical values are secondary to the profession’s accepted standards. Given the importance of conscience to ethical care provision, conscience clauses are embedded in national professional association documents (Canadian Medical Association, 2016; Canadian Nurses Association, 2017b).

Although there is no requirement that Canadian physicians or NP must provide MAID, there is an expectation that physicians and NPs follow their provincial or territorial regulatory policies and guidelines when disengaging from care. In Saskatchewan, the physician must (a) not abandon the patient, (b) treat the patient with dignity and respect, (c) provide sufficient resources to make informed choices and access all care options, (d) arrange timely access to another physician or resources and advice, and (e) provide the patients relevant information and chart when authorized by the patient to do so (College of Physicians and Surgeons of Saskatchewan, 2018). NPs must (a) refer the patient to a physician, NP, or to a designated contact person to provide MAID if requirements are met, and (b) care for other health needs until care is provided by another HCP (Saskatchewan Registered Nurses Association, 2016).

Before MAID legalization, 63% of the Canadian physicians who responded to a Canadian Medical Association (CMA) survey would “refuse outright” to participate in assisted dying (Vogel, 2015). The survey also captured physicians’ opinions regarding what should be done if physicians did not participate in assisted dying: 19% stated that physicians should refer a patient to a colleague, 17% stated that physicians should refer to an independent third party, 17% stated that physicians should refer to a medical administrator, and 29% stated that they should not have to do anything (Vogel, 2015). Some nonparticipation may stem from the belief that MAID violates the Hippocratic Oath, religious convictions, or professional ethics (Canadians for Conscience, 2018).

There are challenges in operationalizing conscience clauses. HCPs should not be forced to participate in MAID, yet there is “disagreement about what this means” (Vogel, 2015, p. E409). Brindley (2017) highlighted concerns that HCPs could use CO to avoid time-consuming or emotionally draining patients, and Lachman (2014) stressed the importance of distinguishing CO from self-interest, discrimination, or prejudice. HCPs with a CO to MAID are required to refer the patient to another HCP; however, MAID referral processes are often imprecisely defined and vary significantly across provinces (Brindley, 2017). In addition, some HCPs view a referral as the moral equivalent to providing MAID (Canadian Medical Protective Association, 2019). However, a recent court decision supported the contrasting position and highlighted that patients would suffer harm without an effective MAID referral (Strathy et al., 2019). In contradistinction to the often dominant legal and rights-based discourse found in CO discussions is a relational decision-making approach (Heilman & Trothen, 2020) that focuses on open and authentic communication amid moral uncertainty within teams of HCPs to seek the best possible patient outcomes (Deutscher, 2016). This approach manages differences in conscience in a way “that does not heavy-handedly subvert one party’s values and moral reasoning for that of the other” (Heilman & Trothen, 2020, p. 127).

## Theoretical Frameworks

Social contract theory (SCT) guided the conceptualization of this research project. Numerous health professions have utilized SCT to consider the social relations, obligations, and conditions under which HCPs carry out their functions and outline the reciprocal rights and responsibilities of HCPs and patients (Rochelle, 1983). Waugh (1993) highlighted that social contracts evolve as laws and professional standards change, as individuals’ needs or expectations advance, or as society diversifies. MAID legalization brought a shift in the social contract of EOL care. Consequently, HCPs need to integrate these evolving rights and responsibilities into their practices, seeking a balance among beliefs and values, the law, practice context, and patient care requests.

Ruggiero’s (2012) approach to moral dilemmas and decision-making also guided this research project. Ruggiero (2012) proposed three decision-making standards to support analytical and objective discourse among individuals. Within this approach, an individual makes decisions while considering the standards of consequences, obligations, and moral ideals. Consequences are the effects of the action on everyone involved (Ruggiero, 2012). Consequences could be beneficial or harmful, physical or emotional, immediately obvious or evident over time, intended or unintended, clearly visible or subtle, and complex or pinpoint. Obligations are influenced by relationships with others and include formal and professional responsibilities and can take the form of friendship, colleagueship, citizenship, or business obligations. Finally, moral ideals are identified as concepts that help individuals achieve respect for others and encompass ethical (i.e., prudence, temperance, justice, and fortitude) and religious values (i.e., faith, hope, and charity).

With MAID as an EOL care option, the expectations of the relationship between patients and HCPs in the existing social contract of care were altered. As a result, HCPs contemplate the consequences, obligations, and ideals relative to these expectations, informing their participation and practice threshold within this new care area. Alternative mechanisms to support the social contract of care may be required to support patients and HCPs relative to their participation threshold.

## Methods

This research is grounded in a constructivist/interpretivist paradigm and used an interpretive description methodology. Within this grounding, there are multiple, sometimes conflicting, socially constructed realities (Ponterotto, 2005). These realities are elicited through interaction between the participants and the researcher and may change as individuals evolve or become more informed (Guba & Lincoln, 1994). Therefore, our research interpretations are specific to the time, participant, and research team context. An interpretive description methodology can support the development of health-related knowledge that is capable of informing and influencing clinical practice through the application of sound qualitative methodology, and it recognizes the researchers’ expertise in the selection of research techniques and approaches (Thorne, 2016). Furthermore, interpretive description accounts for the researchers’ context, the setting, and the participants, and that the data interpretation occurs with the lens of the research team.

The positionality and reflexivity of the research team are essential in an interpretive description methodology. The coauthors and a doctoral committee support the first author. The first author is a registered nurse (RN) with experience in urban, rural, and remote nursing settings. She is currently a doctoral candidate who works as a nurse educator and has an emerging program of research in EOL care and MAID. The second author is an RN, the third author is a physician, and both are professors in the College of Medicine and co-supervise the first author. The fourth author is a gerontologist and associate professor in the School of Public Health. Collectively, they have significant research programs in EOL care, MAID, aging, and program and policy evaluation. The authors met throughout the research process to discuss their underlying and emerging thoughts and opinions to support reflexive processes.

## Methods

### Sampling Strategy

Potential participants were provincially licensed physicians and NPs who self-identified as not participating in formal MAID processes. Specifically, this included participants who (a) were reluctant to engage in MAID-related processes, (b) would decline participation in any aspect of MAID, or (c) identified they did not know how they would respond to a potential patient’s request for MAID assessment or provision. We excluded HCPs who worked exclusively with patients aged under 18 years as this patient group is ineligible for MAID. We aimed to include HCPs who worked in urban, rural, and remote areas and general and specialty practices, and we sought diversity within participants’ gender, age, years of practice, and faith/spirituality background within this purposeful sample. Furthermore, we considered that our sample size should be adequate to represent the experiences of a diverse group of participants, which would contribute meaningful results through responsible analysis (Patton, 2015).

We used three approaches for participant recruitment. First, the provincial health authority, the physician and NP regulatory bodies and professional associations, the cancer agency, the medicine and nursing faculties of the universities, and the division of northern medical care distributed an invitation to participate. This invitation was distributed either by emailing an invitation letter or using ethics board–approved posters and social media scripts, which included pertinent study information such as participant inclusion criteria. Second, we used snowball sampling to augment our recruitment and asked consenting individuals to forward the request for participation through their respective networks. Finally, the doctoral committee members sent the letter of invitation through their networks. Potential participants contacted the first author (the interviewer) to confirm their research participation eligibility and determine a mutually agreeable time and interview modality (in person, telephone, or WebEx). The participants received the information and consent form in advance of the interview and verbal consent was obtained on the interview recording. The first author confirmed that consent was collected on the written consent form, and the participants also confirmed consent on the online contextual information data collection tool.

### Sources of Data and Data Collection

Multiple sources of data were collected and included (a) participant contextual data, (b) interview data, and (c) interviewer reflective and field note content. First, the participant’s contextual data, including gender, age, marital status, the significance of faith, religion or spirituality, belief system, professional group, specialty practice area, years in practice, location of practice, the proportion of patients with a life-limiting illness, and whether an actual or hypothetical MAID request informed their interview responses, were collected on a secure university-provided survey platform. We sent the online link to the participants through email, and they completed it in advance of, or during, the interview. Second, we collected data through a semi-structured interview that utilized vignettes and open-ended, exploratory, and probing questions. The use of vignettes would support exploring the participants’ decision-making processes (Evans et al., 2015), attitudes, perceptions, and beliefs (Hughes & Huby, 2002). We designed the vignettes to address different aspects of participation, including clinical processes (providing information and emotional support, formal MAID assessment, and formal MAID provision), MAID discussions with colleagues, and MAID continuing education (Supplemental File 1). The vignettes were crafted from case histories and the research team’s practice experiences and then vetted by two NP and two physician field experts for suitability to support validity. We invited the participants to respond to the short narratives or scenarios and followed-up with direct, exploratory, or clarifying questions. After four interviews, the research team reviewed the interview data to ensure that the vignettes supported the elucidation of the research objective. No vignette adjustments were deemed necessary. After each interview, the interviewer recorded extensive reflective and field note content to support self-reflexivity in the data collection event and account for its context. Field notes and reflective content included journaling on what would be asked differently, what the interviewer thought was salient, what new lines of inquiry emerged, and how the interviewer felt during the interview process. This supported the iterative interview and data analysis process and informed future interviews. The analysis included all the data as part of the interpretive approach.

### Ethics and Operational Approval

We received ethical approval (REB No. 902) and provincial health authority operational approval (OA-UofS-902) for this research. We provided access information to support programs, given the topic’s potentially sensitive nature, on the information and consent form. In the ethics application, we noted that the researcher and doctoral committee members might have relationships with potential participants. Nonetheless, we did not exclude these participants as these relationships were professional and the health care community in this province is relatively small. The interviews were audio-recorded with the resultant audio file encrypted, transferred, and stored according to the approved ethics board process. The transcriptionist signed a confidentiality agreement and had no access to other study data. We noted that data were accessible to doctoral committee members and procedures for sharing aggregate responses with participants were approved.

### Data Analysis

Data analysis began following each completed interview and continued throughout the data collection process. After the interviews were completed, they were transcribed by one transcriptionist who noted the participants’ filler words and emotional content and redacted the interviewer’s filler words and any identifying information. The participants’ contextual data were summarized using frequencies and percent to account for their personal and practice contexts during data description and interpretation. The first author analyzed the interview transcripts, the field notes, and the reflective content, using reflexive thematic analysis with the support of NVivo12. Inductive coding and constant comparative analysis occurred across the entire data set while reflecting on SCT and Ruggiero’s approach to moral dilemmas and decision-making. These initial patterns of meaning were developed and presented to the participants for member checking and the coauthors before the final data interpretation. Member checking allowed the participants to provide additional reflections, comment whether the data descriptions were realistic and whether the preliminary patterns of meaning were fair (Creswell, 2005), and provided a reflective space for participants (Candela, 2019) as they contemplated their subsequent interview experiences. Two participants responded to the aggregate finding email; no additional data to analyze were provided through this process. These initial patterns of meaning underwent combining, refining, and eventual interpretation and theming (Braun & Clarke, 2019) and were presented to the doctoral committee. The resultant themes were collated with theme definitions and supporting participant data, which formulated the findings’ structure. These documents were cross-checked by the coauthors and presented to the doctoral committee as part of an expert panel analysis check (Thorne, 2016).

### Planning for Quality and Credibility

We prioritized quality and credibility throughout the research project. First, we ensured methodological and method congruence. Second, we accounted for the research team’s positionality and self-reflexivity through the data collection process, using the collection of field notes and reflective content, and team meetings. We established rigor by using multiple data sources, vetting and trialing the vignettes, and using a single transcriptionist and preliminary coder. Rigor was further confirmed by cross-checking the codes to the transcripts by the coauthors, sharing the aggregate findings with participants, and considering the doctoral committee feedback as part of the expert panel analysis review. Finally, we provided detailed descriptions and multiple participant exemplars to support the data analysis.

## Results

Once we interviewed 35 HCPs, our data collection and concurrent analysis ceased. We had broad representation within the contextual data and 452 single-spaced transcript pages and 105 single-spaced field notes and reflective content pages to support the research objective’s exposition.

In response to the vignettes, all HCPs stated that they would direct the patient to discuss MAID with an alternative HCP or refer the patient for continued care. Approximately, 40% (*n* = 14) of HCPs stated that they would not participate in MAID beyond this, whereas other HCPs (*n* = 21) anticipated different nonparticipation thresholds (e.g., discuss MAID as an EOL care option, provide emotional support on the day of death for the patient and family) in the clinical care vignette. We provided the participants’ contextual data in Supplemental File 2 to frame the themed qualitative results. The resultant endogenous factors that influenced nonparticipation themed consistently across the data set.

Participants also discussed their needs relative to nonparticipation in formal MAID processes. As we were open to developing data patterns in the data analysis, these data patterns were subsequently themed and presented as results.

### Factors Influencing Nonparticipation

Numerous factors contemporaneously influenced HCPs’ nonparticipation in formal MAID processes. Some of these factors originated from within the individual HCP, whereas other factors originated external to the individual HCP. *Endogenous factors* are conceptualized as the factors that originated from within the HCP and *exogenous factors* are conceptualized as the factors that originated from beyond the individual HCP. For some HCPs, nonparticipation in formal MAID processes was solely influenced by the endogenous factors, whereas exogenous factors limited nonparticipation for other HCPs. Because the data were so extensive, the exogenous factors will be detailed in subsequent articles.

### Endogenous Factors Influencing Nonparticipation

We themed the endogenous factors influencing HCPs’ nonparticipation in formal MAID processes into eight areas. HCPs’ were influenced by their (a) previous personal and professional experiences, (b) comfort with death, (c) conceptualization of duty, (d) preferred EOL care approaches, (e) faith or spirituality beliefs, (f) self-accountability, (g) consideration of emotional labor, and (h) future emotional impact. As noted previously, 14 HCPs identified how these factors culminated in their decision not to participate in MAID beyond a referral. Other HCPs considered different clinical MAID participation thresholds; however, none would formally administer MAID.

#### Previous personal and professional experiences

Some HCPs shared their personal accounts of living with a life-limiting illness, their personal accounts of watching a close family member with a life-limiting illness die, or their personal accounts of having a family member with a disability. Furthermore, these HCPs shared how these experiences influenced how they viewed MAID as an EOL care option and their participation perspectives.

I had a family member with a disability, and that family member said to me, “the next time I get sick, do not kill me, okay?” He felt obliged to let the record show that he could still do things others could not and was trying to figure out if there was some magic line and make sure he was never over that line. . . If he were in a hospital now, I would not leave him unattended [fearing] a member of the care team would say, “we are cruel to this person. I am going to do him in.”

Other HCPs discussed their professional practice experiences in caring for patients with life-limiting illnesses and EOL care. These experiences significantly shaped their perspectives on formal MAID participation and impacted their participation threshold.

Once you go through a couple of bad [MAID processes], you will see patients unnecessarily suffering while waiting to get everything in place and not being able to have pain medication because they have to be completely cognitive. In the few that I have been sort of, not involved as the person doing it, but involved as the most responsible practitioner and as a support for the family, it wasn’t a positive experience.

I cared for a palliative patient, and I was pushing 2 mg of morphine, and he stopped breathing. I nearly stopped breathing myself. And I know it was not my fault that he stopped breathing. That is what can happen. He just died at that point, and I will never forget that feeling. I cannot. So, no, I could never do anything like that.

#### Comfort with death

HCPs expressed varying degrees of comfort in EOL care and often reflected a general disquiet about death and dying. Furthermore, some HCPs recognized that comfort with dying and death was not inherent to all HCPs and that this comfort with death influenced HCPs’ nonparticipation in formal MAID processes.

[Participation] would be uncomfortable or difficult for me. Umm, umm, viewing the dying process, yeah. Death is difficult, and seeing her die. . . I think it would make me uncomfortable.

It [EOL care] are things that I think it does take a special human to do that comfortably.

#### Conceptualization of duty

Some HCPs described how MAID did not align with their professional practice, their profession’s tenets, and their obligations to the patient and discussed how this influenced their participation in formal MAID processes. Some HCPs were clear that MAID was counter to their conceptualization of professional duty.

It is something that I view as being very separate from me. It is not something that I see as my role in medicine. I don’t see myself as an agent of death. Can I help someone die well? Absolutely. Do I want to be the mechanism of death? No, I do not.

My discussion [with patients] is, “is there anything to address your concerns in terms of your independence and your quality of life?” That is what my role as a doctor is. MAID is counter to my ethos as a doc.

#### Preferred end-of-life care approaches

Participants reflected on how MAID fit within the spectrum of the existing EOL care options. Some HCPs articulated how MAID did not align with their existing EOL care practices and approaches, while others discussed how MAID was not encompassed within their vision of palliative care.

There are so many other options other than “let us just refer to MAID.” I have been in some very beautiful deaths in the palliative care approach. It is not just about the person dying. It is about the experience and what that brings to the family. If you do the MAID program, maybe that’s not going to happen.

My job is to make death a positive experience by controlling symptom management. I am not there to bring on death quicker. I am there to support a natural process. The MAID program is not a natural process, it is the exact opposite of what I do.

#### Faith or spirituality beliefs

Some HCPs shared that MAID was counter to their core spiritual or faith beliefs and discussed their accountability to a higher power. These HCPs further spoke about the importance of aligning their clinical practice with their faith or spirituality beliefs as this provided a source of inner strength and comfort.

To see someone have a peaceful death and go on their terms, I am happy for them, and I am good with that. But when it comes to if it was me actually administering something to take a life? You know, you kind of think about your own demise. When I get up to the pearly gates, how is that going to be viewed?

#### Self-accountability

Some HCPs discussed their self-accountability, including their need to feel at peace and account for their practice and participation choices in MAID. This self-accountability also encompassed being assured that participating in formal MAID processes was the right thing for them to do.

It is different knowing that someone has died in your care and knowing that you ended that life. It is really, it comes down to you are the person that did it. And, I am not ready to accept that right now.

It is such a paradigm shift. . . to actually be there as the one pushing the syringes, like, that I get stuck on. I just need to think about it a little more, yeah. I would have to be incredibly clear in myself, in my soul, and my brain that what I am doing is the right thing to do.

#### Consideration of emotional labor

Many HCPs discussed the emotional labor or the management of feelings (Grandey, 2000) of potential MAID participation and articulated how this consideration of emotional labor influenced their nonparticipation in formal MAID processes. Some HCPs anticipated isolation, guilt, or grief relative to formal participation in MAID. Others contemplated the emotional work of supporting patients and families while processing their own emotions. Other HCPs articulated how their participation perspectives were influenced by their belief that participation in formal MAID processes would compound the moral distress already present in their health care environments.

I think it [participation] would be a train wreck on my part. I do not think I could be like, “okay, I am going to support this.” Like, I couldn’t support it and just sit back and provide emotional support. I almost feel guilty by association.

That sounds very hopeless, but that is my true and honest belief. We already have so much ethical and moral distress, to put ourselves in that [MAID participation] situation, I just can not see it happening.

#### Future emotional impact

Some HCPs considered the future emotional impact of participation in formal MAID processes on themselves and others. They identified potential concerns such as posttraumatic stress disorder, HCP burnout, and the emotional impact of provider isolation. This concern for their future emotional well-being and the subsequent impact on their ability to provide care influenced their nonparticipation perspectives.

I would be worried about physician burnout. . . I think it [MAID] could be harder emotional work than one foresees it being at the start. That would be something that would concern me.

I would like to see the data that comes forward in the next five or ten years on these practitioners who drive around from place-to-place, just to give these provisions to people. I would like to see some data about whether any psychological adversity occurs or if there is any evidence of PTSD.

Some also identified a concern that MAID participation would impact the meaning of caring for individuals and families at the EOL. They further discussed their apprehension that this would impact the quality of patient and family EOL care encounters.

If a person is just doing end-of-life, umm, just doing MAID, I suppose, it might remove the significance of it [MAID participation] for them. And if they do not have that sense of significance anymore, that will affect the interaction, I think, and the experience for the patient.

### Health Care Professionals’ Professional Needs

Within the interviews, two themes emerged regarding HCPs’ professional needs relative to the endogenous factors. HCPs identified a need for (a) clear care pathways, and (b) safe passage.

#### Clear care pathways

All HCPs stated they would support the continuation of care by directing the patient to discuss MAID with an alternative HCP or a direct referral. However, few HCPs could articulate the current referral processes and expressed confusion and uncertainty in where they would go for this information. Other participants identified that HCPs and patients would have challenges obtaining accurate MAID information and achieving seamless care when care pathways were uncertain. This need for clear care pathways was especially crucial, considering recent health delivery reorganization and the MAID program’s newness and evolving nature.

So, I think initially, in each of the health regions, there was a contact. But, that information was really hard to find. Who do you call now?

Not even knowing the name of an assessor or provider to collaborate with is a problem. Unless you happen to know that assessor or provider personally, like through your practice, then that information is not even made known. Which I think is unfortunate because it is really then up to patients to seek that information on their own.

#### Safe passage

Some participants articulated that they were hesitant to bring up their opinions on MAID for fear of losing esteem with their colleagues. Others described the discourse of broaching and discussing their MAID nonparticipation with their colleagues. Furthermore, other HCPs identified a need to feel secure and empowered to dialogue about their nonparticipation in MAID processes, with managers, professional bodies, patients, and families without fear of reprisal or disdain.

Conversations with peers and colleagues are uncomfortable and polarized. . . people I know go, “that [nonparticipation] is wrong.” It is not wrong! It is not wrong! Choose your language appropriately. . . what is right for me is not necessarily right for you, and mind your own business, right?

I have a colleague who inserted a PICC line and ordered an x-ray. He went back to check the x-ray before telling them to go ahead and use the line. He could not find the x-ray. He went to the ward and could not find the patient. The line he had inserted had been used to kill the patient. He had no understanding that was what was going to be done, and it rocked him. He said, “I want nothing to do with putting in lines to kill people,” and the manager said, “Suck it up buttercup, it is not your job to question, it is your job to put lines in.”

Other participants identified the need for respectful, safe, and transparent processes to support their disengagement from MAID and recognition that their nonparticipation perspectives were valuable. Collectively, these perspectives were themed as the need for safe passage.

I am very aware that there are some folks in the system that are just waiting for the old dinosaurs [the HCPs who do not participate in MAID] to disappear. I think there needs to be a very clear articulation of appreciation for different perspectives and not just tolerance and accommodation.

## Discussion

### Reconciliation

**Figure 1.**
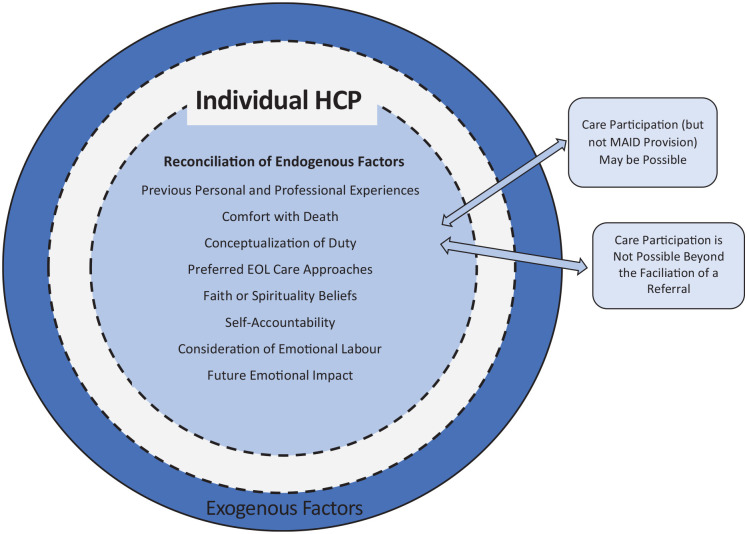
Endogenous factors influencing HCPs’ nonparticipation in formal MAID processes. *Note*. HCPs = health care providers; EOL = end-of-life.

HCPs’ contemplation of the endogenous factors is conceptualized as reconciliation. Reconciliation is not an agreement or acceptance of MAID as an option, nor is it an expression of a willingness to participate in MAID processes. The reconciliation process harmonizes the endogenous factors with the HCP’s formal MAID participation threshold relative to their current clinical practice. By reconciling the endogenous factors, some HCPs anticipated that care participation beyond a referral’s facilitation was not possible. Whereas other HCPs reconciled the endogenous factors and anticipated different MAID participation thresholds (while yet identifying as being unable to participate in provision) in the clinical care vignette. A visual representation of the results is in Figure 1. As noted previously, the exogenous factors will be noted in subsequent articles.

### Integration of Theoretical Frameworks

HCPs consider multiple factors regarding their nonparticipation in formal MAID processes. These include their (a) previous personal and professional experiences, (b) comfort with death, (c) conceptualization of duty, (d) preferred EOL care approaches, (e) faith or spirituality beliefs, (f) self-accountability, (g) consideration of emotional labor, and (h) future emotional impact. Considering Ruggiero’s (2012) framework, the factors influenced how HCPs consider and eventually reconciled their obligations and moral ideals relative to the consequences of participation. As HCPs engage with and integrate new personal and professional experiences, their conceptualization of the other factors may shift. Hence, their participation perspectives may also change. This illustrates the dynamic interplay between the endogenous factors influencing HCPs and suggests that, for some HCPs’, their perspectives on formal participation in MAID processes may evolve.

Moral ideals promote notions of excellence and, for “highly ethical people, the line between obligations and ideals tends to be blurred. . . [as] people tend to view ideals as obligations that they hold themselves for meeting” (Ruggiero, 2012, p. 122). The blurring of obligations and ideals may be particularly pertinent for HCPs, given their responsibility to ethical codes, which was confirmed by how HCPs’ ethics, EOL care approaches, and professional duty beliefs were intertwined. In the reconciliation of ideals and obligations, some HCPs were most influenced by their ideals, which resulted in HCPs’ nonparticipation in formal MAID processes and a desire to fulfill the social contract obligations through alternative means. Ruggiero’s standard of consequences was evident by the depth to which HCPs contemplated their self-accountability, the emotional labor of participation, and identified concerns regarding the future emotional impact of participation. According to Ruggiero (2012), individuals will choose actions that have favorable consequences (or avoid negative consequences) while honoring the weighting of their obligations and ideals. In our research project, in alignment with our sampling criteria, these choices resulted in all participants avoiding all participation in formal MAID processes beyond the facilitation of a referral.

### Practice Implications

Considering these results, we bring forward system-level recommendations, including opening the discourse, referral pathways attentive to moral space, safe passage grounded in respect, and attention to emotional labor.

#### Opening the discourse

HCPs may not participate in formal MAID processes due to reasons of conscience. Conscience is “an internal moral decision-making process that holds someone accountable to their moral judgment, and for their actions” (Lamb et al., 2019, p. 1338). However, as Wicclair (2011) noted, not all nonparticipation is conscience-based, and nonparticipation may derive from self-interest (i.e., concern for individual health and safety) and individual HCP’s consideration of professional integrity (i.e., understanding and application of clinical and professional norms and standards). Thus, in alignment with Wicclair’s description of non-conscience-based refusals, there are non-conscience-based limiters to participation in formal MAID processes within our themed results.

Our regional research findings align with the emerging literature regarding nonparticipation in MAID in Quebec and international research in nonparticipation in assisted dying. Canadian research identified conscience-based and non-conscience-based reasons to refuse to perform MAID; conscience-based reasons included “moral” and “religious“ grounds, and non-conscience-based reasons encompassed “emotional reasons,” capacity, and competency reasons (Bouthillier & Opatrny, 2019, p. 1217). Internationally, HCPs’ refusals to participate in assisted dying were based on conscience-based reasons such as “religious opposition,” “personal values and ethics,” and “strong moral convictions” as well as non-conscience-based reasons, such as considerations of legal and professional risk, patient factors, personal competence, their preference for other care options, emotions, and fear (Clymin et al., 2012; Willems et al., 2000). Collectively, we recognize that ethical, religious, or core moral beliefs (conscience-based factors) and non-conscience-based factors both influence HCPs’ nonparticipation in formal MAID processes. Thus, we bring forward the need for two separate yet overlapping concepts: CO to MAID and nonparticipation in MAID for reasons other than conscience, both impacting the social contract of care.

#### Referral pathways attentive to moral space

HCPs need referral pathways to facilitate MAID access and support the social contract of care. Referral pathways will mitigate the tensions that can occur when one party’s expectations in the social contact are ignored or “responded to in a way that is thought to be inappropriate” (Waugh, 1993, p. 1321). Actualizing referral pathways for MAID access is essential yet complex. Accommodating conscience provides a “moral space” that allows HCPs to practice without compromising their moral integrity (Wicclair, 2011, p. 25). Moral distress, or the emotions and attitudes that arise in response to being involved in morally undesirable situations, occurs when conscience is not accommodated (Campbell et al., 2016). Moral distress, in turn, can harm HCPs’ well-being and can impact job retention (Lamiani et al., 2017). Thus, care referral pathways should facilitate timely and unencumbered access to care while being attentive to the moral concerns of HCPs. As some HCPs consider their complicity in and shared responsibility for morally objectionable practices, referrals for MAID care may be challenging. As Trigg (2017) explained, “it should not be the responsibility of any professional to help someone on the first steps to something if they are not willing to go with that person the rest of the way” (p. 43). Therefore, a relational and compromising approach would be to have multiple MAID access pathways, including HCP-initiated *and* patient-initiated referrals.

HCP-initiated referrals may be imperfect in all practice areas due to missing or sparse clinical information and variation in referral expectations and processes (Canadian Medical Association, 2014). Therefore, mechanisms to optimize, expedite, and clarify the referral process, including those for MAID, are essential to support the social contract of care. Patient-initiated referrals are based on assumptions that patients know of their ability to self-refer and have the agency to do so. However, patients at the end of life are vulnerable, as they live with their care burdens, have restricted activities, fears, insecurities, loneliness, and the prospect of facing death (Bosch-Barrera & Bota, 2016). Patients may also believe that HCP-initiated referrals are required, given the traditional “gatekeeping” and “patient navigating” roles of HCPs (Strathy et al., 2019). Patient-initiated referrals are also imperfect as they may lack the essential clinical information required by the receiving assessors. Despite these concerns, patient-initiated referrals do provide an additional pathway for patients to access care. Both HCP-initiated and patient-and-family-initiated referrals were options for care continuation at the time of our research, yet many HCPs were unaware of this. Thus, just as crucial as the need to have multiple referral pathways is the need to communicate their availability to patients, patient’s families, patient advocates, and all health care team members. Only when all parties within the social contract are aware of the referral pathways and are empowered to use them will the social contract truly be fulfilled.

#### Safe passage grounded in respect

Safe passage is to “go somewhere without being attacked,” or a protection “offered to someone in danger or who is traveling through a dangerous place” (Collins, 2021, paras. 1–2) or creating a caring “environment in which people are assured that it is safe” (Lazenby et al., 2014, p. 118). Within the context of these findings, safe passage, or the ability to work within one: moral space in safe and satisfying work environments, is required by HCPs as they traverse the terrain of nonparticipation in formal MAID processes. HCPs, care teams, and administrators must have authentic, respectful, and open conversations grounded in relational ethical decision-making to support HCPs who do not participate in formal MAID processes. This caliber of discourse (a) allows HCPs to reflect on their practice demands and the laws, rules, and policies that impact their practice; (b) respects the moral agency of those who hold dissenting views; and (c) fosters an examination of the reasons for dissent (Weinstock, 2014). Furthermore, health systems must move beyond policy-level support for CO to actually “identifying how the facility and staffing logistics are managed concerning MAID, and how, when, and to whom objection will be communicated to ensure the continuation of safe care” (Brown et al., 2020, p. 7). There is very little research that has explored how HCPs make their objections known and very little research that has identified the consequences to HCPs when declaring a CO on HCPs (Lamb et al., 2017), so clarifying and evaluating these processes are especially crucial.

#### Attention to emotional labor

HCPs are considering the emotional labor of formal MAID process participation. Emotional labor in EOL care is often overlooked (Brighton et al., 2019). Caring for dying persons and their families is a source of emotional distress, and HCPs’ grief may be suppressed, prevented through emotional detachment, or may “spillover” into their private lives (Funk et al., 2017, p. 2216). EOL care is complex (Ferrer et al., 2016), is challenged by various communication barriers (Ethier et al., 2018), and how HCPs view EOL teamwork (Goldsmith et al., 2010). MAID assessors and providers identified rewarding elements to care participation and care participation challenges. MAID has been viewed as a calling and as an act of service (Beuthin, 2018), and those who participate in MAID noted its significant responsibility, how “meaningful the practice of MAID was to them and their patients,” and the gratitude extended by patients and families (Shaw et al., 2018, p. e397). On the contrary, care participation stressors were noted, including isolation, lack of support, challenging relationships with objecting colleagues, sacrifices to personal time, working with institutions with a CO, denying patients who did not qualify for MAID, and the grief of family and friends (Khoshnood et al., 2018; McKee & Sellick, 2018; Shaw et al., 2018). Thus, participants in our project were justified when anticipating emotional labor in formal MAID processes; in addition, the process of reconciling the availability of MAID relative to their formal participation inherently also involved emotional labor. In agreement with Brighton et al. (2019), it is vital to acknowledge EOL care’s emotional labor (which includes participation in formal MAID processes) and normalize the need for HCPs’ support. We further extend the need to acknowledge and support the emotional labor of reconciliation.

### Areas of Future Research

Future research could evaluate whether there are variations in the endogenous factors across other subgroups or regions of Canada. As this study occurred approximately 3 years after MAID legalization, a follow-up study could ascertain whether the factors identified as influencing nonparticipation in MAID change or evolve the longer MAID is legally available. With increased utilization of patient-initiated referrals, research to explore the patient and family perspectives on accessing care through this manner would help understand their contributions to the social contract of care. Future inquiry into HCPs’ and patients’ perspectives on the reciprocal rights and responsibilities in the MAID social contract of care would provide insight into care provision, as this social contract evolves. Finally, it is highly essential to examine the necessity and efficacy of practice and emotional supports for HCPs who participate in MAID processes and evaluate the long-term impact of participation in formal MAID processes on HCPs’ mental and emotional health.

### Limitations

Our research team interpreted the participants’ experiences and perspectives in our geographic location at a specific point in time; thus we have provided rich contextual information to assess the findings’ transferability. Although we had a deep, rich data set and a significant degree of code redundancy, we acknowledge that additional participant perspectives may be discovered in alignment with our research paradigm and methodology. As several participants had not experienced an actual patient request for MAID, their responses were hypothetical. Finally, there is little available Canadian research in this area to position our findings, and the referenced international research may not approximate Canadian culture, laws, and health care delivery.

## Conclusion

The factors influencing HCPs’ nonparticipation in formal MAID processes are complex, diverse, and interwoven. In exploring these factors, we identified two separate yet overlapping concepts; CO to MAID and nonparticipation in MAID. To support the evolution of social contract relative to MAID, HCPs require referral pathways attentive to the moral space and safe passage. Having both HCP-initiated and patient-initiated referral pathways in place may support this; however, the pathways’ availability and the process must adequately be communicated to all stakeholders. Furthermore, there must also be recognition and support for the emotional labor of reconciliation and MAID nonparticipation. Finally, health systems should support HCPs’ CO at the point of care by clearly identifying the mechanisms to disengage from care for HCPs, and openly discuss, with appreciation, the diversity of MAID participation perspectives.

## Supplemental Material

sj-docx-1-qhr-10.1177_10497323211008843 – Supplemental material for “What Is Right for Me, Is Not Necessarily Right for You”: The Endogenous Factors Influencing Nonparticipation in Medical Assistance in Dying Click here for additional data file. Supplemental material, sj-docx-1-qhr-10.1177_10497323211008843 for “What Is Right for Me, Is Not Necessarily Right for You”: The Endogenous Factors Influencing Nonparticipation in Medical Assistance in Dying by Janine Brown, Donna Goodridge, Lilian Thorpe and Alexander Crizzle in Qualitative Health Research

sj-docx-2-qhr-10.1177_10497323211008843 – Supplemental material for “What Is Right for Me, Is Not Necessarily Right for You”: The Endogenous Factors Influencing Nonparticipation in Medical Assistance in Dying Click here for additional data file. Supplemental material, sj-docx-2-qhr-10.1177_10497323211008843 for “What Is Right for Me, Is Not Necessarily Right for You”: The Endogenous Factors Influencing Nonparticipation in Medical Assistance in Dying by Janine Brown, Donna Goodridge, Lilian Thorpe and Alexander Crizzle in Qualitative Health Research
