# Supplementary material for: “What Is Right for Me, Is Not Necessarily Right for You”: The Endogenous Factors Influencing Nonparticipation in Medical Assistance in Dying
Source: Qual Health Res. 2021 May 3;31(10):1786–800. doi: 10.1177/10497323211008843 (PMC8446887; doi:10.1177/10497323211008843)
Supplement: sj-docx-2-qhr-10.1177_10497323211008843 – Supplemental material for “What Is Right for Me, Is Not Necessarily Right for You”: The Endogenous Factors Influencing Nonparticipation in Medical Assistance in Dying [file sj-docx-2-qhr-10.1177_10497323211008843.docx]

Supplemental File 2: Contextual Data of Participants (N=35)

| **Item** | **Variable** | **Participants Who Would Not Participate Beyond a Referral n = 14 (40%*)** | **Participants Who Would Provide More than a Referral, but not Formally Administer MAID**  **n = 21 (60%*)** |
| --- | --- | --- | --- |
| Gender: | Female  Male | 9 (64%)  5 (36%) | 14 (67%)  7 (33%) |
| Marital Status: | Single/Never Married  Married/Domestic Partnership  Divorced | 1 (7%)  13 (93%)  - | -  17 (81%)  4 (19%) |
| Age (years): | 25-34  35-44  45-54  55 and older | 3 (21%)  4 (29%)  2 (14%)  5 (36%) | 2 (10%)  5 (24%)  8 (38%)  6 (29%) |
| Faith, Religion, Spirituality, Significance: | Extremely Significant  Very Significant  Significant  Neutral  Not Significant | 7 (50%)  3 (21%)  2 (14%)  2 (14%)  - | -  5 (24%)  2 (10%)  11 (52%)  3 (14%) |
| Which of the following describes your belief system? | Protestant  Non-denominational Christianity  Agnostic/Atheist  Islam  Roman Catholic  Did not disclose/Other | 4 (29%)  3 (21%)  -  1 (7%)  5 (36%)  1 (7%) | 3 (14%)  3 (14%)  4 (19%)  -  8 (38%)  3 (14%) |
| Professional Affiliation: | Nurse Practitioner  Physician | 6 (43%)  8 (57%) | 12 (57%)  9 (43%) |
| Years in Practice: | 1-9  10-19  20-29  30-39 | 6 (43%)  3 (21%)  4 (29%)  1 (7%) | 6 (29%)  8 (38%)  4 (19%)  3 (14%) |
| Location of Practice:** | Large Population Centre  Medium Population Centre  Small Population Centre  Rural area | 5 (36%)  1 (7%)  3 (21%)  5 (36%) | 11 (52%)  2 (10%)  6 (29%)  2 (10%) |
| Primary Work Area: | Family Medicine/Primary Care  Specialty Practice Areas*** | 8 (57%)  6 (43%) | 13 (62%)  8 (38%) |
| Percentage of Patients on Caseload with Life-Limiting Illness: | 0-19%  20-39%  40% or more | 8 (57%)  4 (29%)  2 (14%) | 15 (71%)  4 (19%)  2 (10%) |
| Responses in the interview were informed by: | An actual patient request  A hypothetical request | 4 (29%)  10 (71%) | 5 (24%)  16 (76%) |

* May not add to 100% due to rounding.

** The Statistics Canada definitions were provided to the participants to support selection.

*** Including, but not limited to, emergency medicine, internal medicine, anesthesiology, surgery, critical care, psychiatry, and physical and rehabilitation medicine.
